# Electron Beam Irradiation: A Method for Degradation of Composites Based on Natural Rubber and Plasticized Starch

**DOI:** 10.3390/polym13121950

**Published:** 2021-06-11

**Authors:** Elena Manaila, Gabriela Craciun, Daniel Ighigeanu, Ion Bogdan Lungu, Marius Dumitru, Maria Daniela Stelescu

**Affiliations:** 1 Electron Accelerators Laboratory, National Institute for Laser, Plasma and Radiation Physics, 409 Atomistilor St., 077125 Măgurele, Romania; elena.manaila@inflpr.ro (E.M.); daniel.ighigeanu@inflpr.ro (D.I.); marius.dumitru@inflpr.ro (M.D.); 2 Horia Hulubei National Institute for R&D in Physics and Nuclear Engineering, Multipurpose Irradiation Facility Center—IRASM, 30 Reactorului St., 077125 Măgurele, Romania; ion.lungu@nipne.ro; 3 National R&D Institute for Textile and Leather—Leather and Footwear Research Institute, 93 Ion Minulescu St., 031215 Bucharest, Romania; dmstelescu@yahoo.com

**Keywords:** natural rubber, filler, plasticized starch, electron beam, degradation

## Abstract

Polymeric composites based on natural rubber (NR) and plasticized starch (PS) obtained by peroxide cross-linking have been subjected to electron beam irradiation in order to investigate their degradation. The amount of PS ranged from 10 to 50 phr and the irradiation dose from 150 to 450 kGy. Irradiation was performed in atmospheric conditions using a linear electron accelerator of 5.5 MeV. Changes in chemical, physical, structural, and morphological properties of composites were correlated with variables, such as PS loading and irradiation dose. Thus, mechanical properties, gel fraction, cross-linking degree, water uptake, weight loss in toluene/water were compared with those obtained before irradiation. The changes in structure and morphology were studied by Fourier Transform Infrared Spectroscopy and Scanning Electron Microscopy. Both PS loading and irradiation dose were found to be responsible for the degradation installing. Moreover, it has been shown that at the dose of 450 kGy, chain scission is dominant over cross-linking.

## 1. Introduction

Huge and valuable sources of rubber are wasted in the absence of recycling. After vulcanization in the presence of stabilizers and other additives, cross-links between the rubber chains are formed so that its natural degradation process is slow [1,2,3]. By applying external energy, the three-dimensional cross-linked rubber network breaks down into lower molecular weight fragments, producing a physical recovery of rubber waste by using these fragments as a non-reinforcing filler [2].

The energy sources for polymer degradation can be thermal, mechanical, photochemical, biological, chemical, or from ionizing radiation, which excites active species such as free radicals, ions, and molecules and can significantly modify the molecular structure of the irradiated material. Ionizing irradiation of organic polymers induces molecular chain branching and cross-linking, which increase the molecular weight of the polymer, but also chemical degradation or scission leading to breakage of the main chains of the macromolecule. Thus, the number and nature of the double bonds change and a decrease of the molecular weight occurs, as well as oxidation of the polymer [4,5]. Depending on the polymer initial molecular structure and morphology, one of these processes that coexists during irradiation, may become predominant. Therefore, the degradation of polymers occurs due to the induced free radical processes in material during irradiation. The higher the dose absorbed by the irradiated material, the greater the free radical degradation capacity. Also, the irradiation dose and dose rates influence the degradation phenomena, as follows: at high values the process is concentrated in the external layers, while at low dose rate, degradation includes larger parts of the irradiated polymer [6]. Two kinds of ionizing radiation are used in polymer processing: gamma rays emitted by radionuclides such as isotopes of cobalt-60, which deeply penetrate materials, but are characterized by relatively small dose rates (between 0.01–0.1 kGy/min) and high-energy electron beams generated by accelerators that have limited penetration depth, but are characterized by continuity, homogeneity, high dose rates (100–600 kGy/min), and ease of starting and stopping. The characteristics of electron beams favor the oxidation reactions that are responsible for the appearance and development of the degradation process in irradiated material [5].

Natural rubber is an essential raw material naturally occurring in high cis-1,4- polyisoprene and is the basis for obtaining over 40,000 products in industry or in various applications. It is non-toxic and has excellent physical properties [7]. To improve mechanical properties, viscosity, and thermal stability, the most common physical–chemical treatment of rubber is curing by sulphur or peroxides [8]. Peroxide-cured elastomers contain high thermal stable C–C bonds and, therefore, exhibit high-temperature ageing resistance [9]. To improve the cross-linking process, polyfunctional monomers are used [10]. Nowadays, huge efforts are made in order to replace the conventional hazardous active fillers as silica or carbon black with environmentally friendly fillers. Starch is seen as one of the most promising natural biopolymers to develop new NR-based composite materials [11]. With plasticizer agents (glycerol, water, and other polyols), starch acquires thermoplastic properties [12]. Its use pursues, on the one hand, the improvement of the mechanical properties of materials and, on the other hand, the increasing of degradability [13]. By irradiation, the starch macromolecule de-polymerizes and the molecular weight decreases. Also, the alteration of the double helix in the branched regions and crystalline structure occurs [14].

The goal of this paper is to study the degradation of elastomeric composites based on natural rubber (NR) and plasticized starch (PS) cross-linked with peroxide in the presence of Trimethylolpropane trimethacrylate (TMPT). An electron beam of 5.5 MeV at a dose rate of 1.5 kGy/min was used for this purpose. Elastomeric composites containing five PS loading, from 10 to 50 ppm, were investigated before and after electron beam treatment with irradiation doses of 150, 300, and 450 kGy. Mechanical characteristics, gel fraction, cross-linking degree, water uptake, and weight loss in solvents were evaluated by specific analysis and the results were correlated with the changes registered in structure and morphology of the composites in order to highlight the degradation process.

## 2. Materials and Methods

### 2.1. Materials and Samples Preparation

The raw materials that have been used for composites obtaining are presented in Table 1.

Five types of composites containing natural rubber (NR) and plasticized starch (PS) have been obtained based on the recipes presented in Table 2. PS and NR/PS mixtures, obtained as in our previous work [13], were cross-linked using dibenzoyl peroxide in the presence of TMPT, a polyfunctional monomer used here as curing co-agent. 

PS was obtained by mixing at 70 °C, starch (50%), water (20%), and glycerine (30%) for 15 min at 50–100 rpm until the homogeneity was attended. The homogeneous mixture was left to rest for 1 h, then introduced in the oven at 80 °C for 22 h and at 110 °C for another 2 h. Finally, it was left to cool down for at least 16 h in a dry place.

The blends were prepared on an electric roller mixer. The constituents were added in the following sequences and amounts: NR was mixed in the roller mixer for 2 min, PS and glycerine (mixing time between 5 and 30 min), antioxidant (mixing time: 1 min), peroxide and TMPT (mixing time: 1 min). After all the ingredients were added, the blend was homogenized for another 2 min and removed from the roller mixer in the form of a sheet. The sheets were cured using molds and a vulcanization press in order to obtain rubber plates with sizes of 150 × 150 × 2 mm^3^, as required for die punching test specimens. The compression temperature in the molding machine was kept constant at 160 °C, for 20 min at a pressure of 300 kN. Cooling time was of 10 min at 25 °C and 300 kN [13].

Samples prepared as above were irradiated at 150, 300, and 450 kGy in atmospheric conditions and at room temperature of 25 °C, using the ALID-7 electron beam accelerator from the National Institute for Laser, Plasma and Radiation Physics, Magurele, Romania. The nominal values of the electron beam (EB) parameters are as follows: energy −5.5 MeV, peak current −130 mA, mean output power −134 W, pulse repetition −3.75 μs, and frequency −50 Hz [15,16,17]

The irradiation process performance depends on the rigorous control of the irradiation dose and dose rate [18,19]. In our experiments, the dose rate was of 1.5 kGy/min. The primary standard graphite calorimeter was used for radiation dosimetry. In order to ensure equality between the entrance and exit dose in the irradiated sample, and for an efficient use of the electron beam, the penetration depth was calculated according to the following equation [16,19].

*E* = 2.6⋅*t*⋅*ρ* + 0.3 (1)

where *E* (MeV) is the electron beam energy, *t* (cm) is the sample thickness, and *ρ* (g·cm^−3^) is the sample density (in our case, 1 g·cm^−3^). The proper thickness of samples subjected to EB irradiation was calculated as being 20 mm [16,20].

### 2.2. Laboratory Tests

Mechanical tests, sol-gel and cross-link density analysis, and water uptake and weight loss in toluene and water tests were made for all blends (C0–C50) on five samples and the results are the averages of these five measurements. 

#### 2.2.1. Mechanical Characteristics Evaluation

Tensile strength (σ) and specific elongation (ε) were determined using the Material Testing Machine ProLine Z005 from Zwick-Roell, Ulm, Germany. The measurements were done in accordance with DIN EN ISO 527-1. Hardness was measured using a hardness tester according to ISO 7619-1/2011 on 6 mm thick samples.

#### 2.2.2. Sol-Gel and Cross-Link Density Analysis

The sol-gel and cross-link density analysis on samples cross-linked with peroxide and irradiated with EB, were carried out as in our previous works [15,16,17]. In order to determine the gel content in the cross-linked products (gel fraction), the solvent extraction method using toluene was used [15,16,17]. Samples weighed and swollen in toluene for 72 h, were dried in air for 6 days and in a laboratory oven at 80 °C for 12 h in order to completely remove the solvent. Samples were finally, reweighed. The gel fraction was calculated as follows:

(2) $$\mathrm{Gel}\;\mathrm{fraction}=\frac{m_{s}}{m_{i}}\times 100$$

where *m_s_* and *m_i_* are the weight of the dried sample after swelling and the weight of the sample before swelling, respectively [21,22].

The cross-link density (*ν*) was determined based on equilibrium solvent-swelling measurements in toluene and by applying the modified Flory–Rehner equation for tetra functional networks. 

Samples with thickness of 2 mm were initially weighed (*m_i_*) and immersed in toluene for 72 h. Then, the swollen samples were removed and cautiously blotted with tissue paper to remove the solvent in excess before being weighed (*m_g_*) in special ampoules to avoid toluene evaporation during weighing. All samples were dried in air for 6 days and in a laboratory oven at 80 °C for 12 h to completely remove the solvent. Finally, the samples were weighed for the last time (*m_s_*) and the volume fractions of polymer in the samples at equilibrium swelling (*ν_2m_*) were determined from swelling ratio *G* as follows:

(3) $$\nu_{2m}=\frac{1}{1+G}$$

where

(4) $$G=\frac{m_{g}-m_{s}}{m_{s}}\times \frac{\rho_{e}}{\rho_{s}}$$

and *ρ_e_* and *ρ_s_* are the densities of rubber samples and solvent (0.866 g/cm^3^ for toluene), respectively.

The densities of elastomer samples were determined by the hydrostatic weighing method, according to the SR ISO 2781/2010. Through this method, the volume of a solid sample is determined by comparing the weight of the sample in air to the weight of the sample immersed in a liquid of a known density. The volume of the sample is equal to the difference between the two weights divided by the density of the liquid. The cross-link densities of the samples, *ν*, were determined from measurements in a solvent, using the Flory–Rehner relationship:

(5) $$\nu =\frac{Ln(1-\nu_{2m})+\nu_{2m}+\chi_{12}\nu_{2m}^{2}}{V_{1}\left(\nu_{2m}^{1/3}-\frac{\nu_{2m}}{2}\right)}$$

where *V*_1_ is the molar volume of solvent (106.5 cm^3^/mol for toluene), *ν*_2m_ is the volume fraction of polymer in the sample at equilibrium swelling, and *χ*_12_ is the Flory–Huggins polymer-solvent interaction term (the value of *χ*_12_ is 0.393 for toluene) [21,22].

The value of Flory–Huggins polymer-solvent interaction term ($\chi_{12}$) for the natural rubber—toluene system was 0.393 [22,23]. 

#### 2.2.3. Cross-Link and Chain Scission Yields Evaluation

For the quantitative evaluation of cross-linking and chain scission yields of irradiated NR and NR/PS mixtures, were done plots of *S* + *S*^1/2^ vs. 1/absorbed dose (*D*) from the Charlesby–Pinner equation [24,25]

(6) $$S+\sqrt{S}=\frac{p_{0}}{q_{0}}+\frac{1}{\alpha P_{n}D}$$

where, *S* is the sol fraction (*S* = 1-gel fraction), *p_0_* is the degradation density—the average number of main chain scissions per monomer unit and per unit dose, *q*_0_ is the cross-linking density—proportion of monomer units cross-linked per unit dose, *P_n_* is the number averaged degree of polymerization, and *D* is the radiation dose in kGy.

Charlesby–Pinner Equation (6), has been used by many researchers so far for simultaneous determination of the cross-linking and the chain scission reaction yields of polymers being irradiated by ionizing radiation. The increasing of *p*_0_/*q*_0_ ratio is associated with the prevalence of scission reaction against cross-linking, being well known the fact that *p*_0_ represents the degradation degree (average number of chain scissions per monomer unit) and *q*_0_ the cross-link density.

#### 2.2.4. Water Uptake Tests

The water absorption in irradiated NR and NR/PS composites was evaluated as in previous works [15,16,17], in accordance with ISO 20344/2011, by immersion in distilled water until the samples no longer absorbed water.

#### 2.2.5. Weight Loss in Toluene and Water

The NR/PS composites weight loss in toluene was evaluated based on the results obtained after the analysis of gel fraction and cross-linking degree. Samples’ weight loss in water was evaluated based on the results obtained in water uptake tests. Weight equilibrium in toluene was reached after 6 days and after 35 days in water, respectively. After reaching equilibrium, samples were dried in air for 6 days and then in a laboratory oven at 60 °C for 24 h. 

#### 2.2.6. Structural Investigations through Fourier Transform Infrared Spectroscopy

The NR/PS composites structure, before and after EB treatment, was investigated by Fourier Transform Infrared Spectroscopy (FTIR) analysis using the TENSOR 27 (Bruker, Germany) FTIR spectrophotometer. The obtained absorption spectra were the results of 30 scans mediation in the range of 4000–600 cm^−1^, with a resolution of 4 cm^−1^.

#### 2.2.7. Morphological Investigations through Scanning Electron Microscopy

The surfaces of NR/PS composites, before and after EB treatment, were examined by Scanning Electron Microscopy (SEM) technique using the FEI/Phillips scanning electron microscope (Hillsboro, OR, USA). Samples, fractured in liquid nitrogen and sputtered with gold palladium, were placed in aluminum mounts and scanned at an accelerating voltage of 30 kV.

## 3. Results and Discussion

### 3.1. Mechanical Characteristics

**Figure 1:**
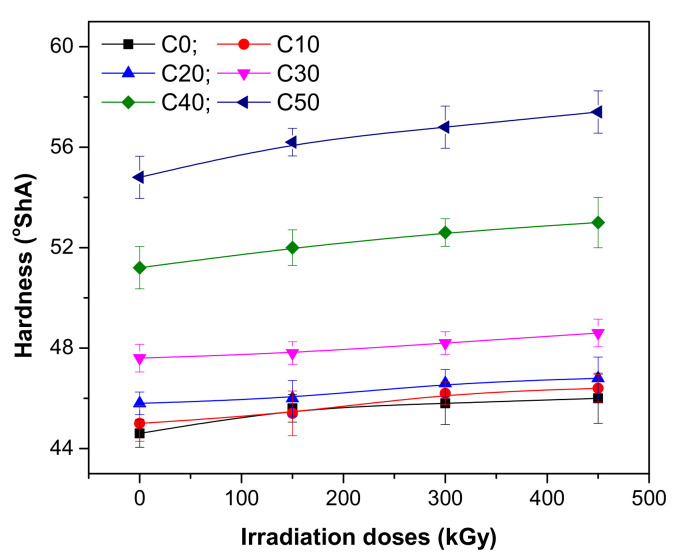
Hardness variation as a function of EB irradiation dose and PS amount.

**Figure 2:**
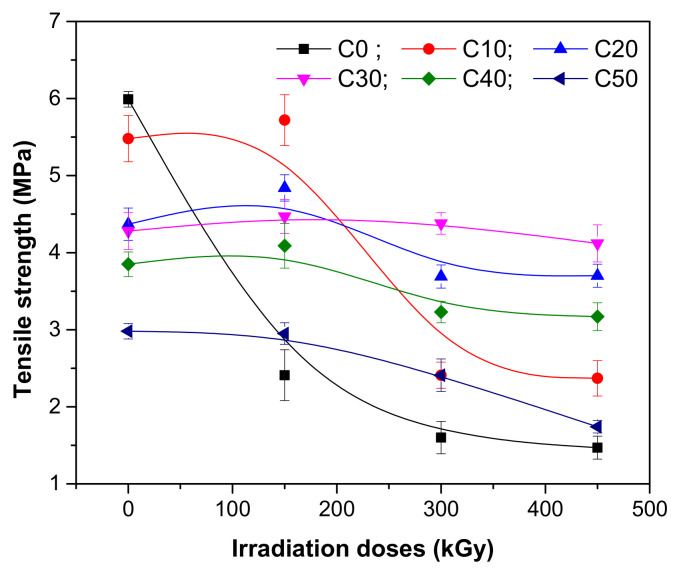
Tensile strength variation as a function of EB irradiation dose and PS amount.

**Figure 3:**
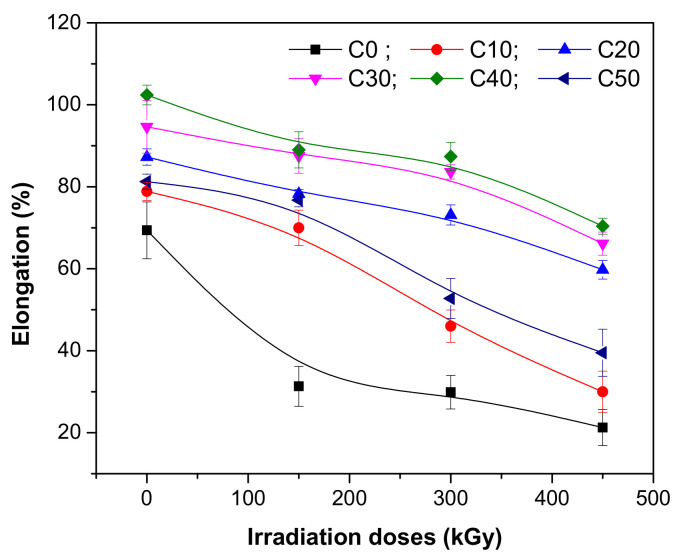
Elongation at break variation as a function of EB irradiation dose and PS amount.

The influence of PS amount and irradiation dose on hardness, tensile strength, and elongation of NR/PS composites were evaluated. The results are presented in Figure 1, Figure 2 and Figure 3. 

The hardness of composites based on polymeric matrix is a measure of their relative stiffness. It is well known that the addition of fillers leads to the increasing of hardness compared to that of the polymer matrix itself [26]. 

The results presented in Figure 1 show the increasing of hardness as the PS amount and irradiation dose increase. The reinforcement effect, due to the addition of 50 phr of PS, was observed even before irradiation: hardness increased from 44.6 to 54.8 °ShA. At the irradiation dose of 450 kGy, the hardness of NR itself increased up to 3.13%. At the same irradiation dose, the hardness of blends containing PS in amounts between 10 and 50 phr were modified as follows: 3.11% (C10), 2.18% (C20), 2.10% (C30), 3.51% (C40) and 4.74% (C50), respectively. The results are associated with the stiffening of the composites due to the reduction of plasticity and flexibility of rubber chains [27].

The tensile strength of a material is a measure of its resistance to breakage, associated also with the maximum elongation until its breakage. As seen in Figure 2, before irradiation, the tensile strength of NR samples decreased as the filler amount increased as follows: from 5.99 MPa to 5.48 MPa for the loading of 10 phr of PS and 2.98 MPa for the loading of 50 phr of PS, respectively. It seems that as the amount of PS increases, its trend is to agglomerate, leading to a faulty interface with the NR matrix. So, the mixture preparation technique, especially in the situation of high loadings, is very important because the appearance of holes in the composite is possible [28,29]. 

The EB irradiation influences the material tensile strength. At the lowest irradiation dose of 150 kGy, the tensile strength of NR/PS samples presents a small increasing that is far from the expected degradation effect. It looks like this irradiation level leads to an improvement of interaction between NR and PS, the polymer chains movement being restricted [30,31] by the presence of filler and improved adhesion [27,32].

At the irradiation dose of 150 kGy, the cross-linking reaction—potentially unfinished in the NR/PS curing with peroxide—is continued. The statement is supported by the initial tendency of tensile strength to increase at this irradiation dose, especially of NR and C10 samples and by the results obtained in gel fraction and cross-link density analysis. More than that, the small amount of PS in C10 made that its behavior to be closer to that of NR compared to that of the other samples (C20–C50).

As the radiation dose increased to 300 and 450 kGy, respectively, a dramatic decrease of tensile strength was observed. The decreasing of the NR tensile strength with the irradiation dose was as follows: less with 59.76% at 150 kGy, with 73.28% at 300 kGy, and with 75.46% at 450 kGy. For the NR samples loaded with 10 ppm of PS, the decreasing of tensile strength with the irradiation dose was a little bit smaller (less with 56.02% at 300 kGy and with 56.75% at 450 kGy). For the NR samples loaded with 50 ppm of PS, the percentages were the smallest obtained (19.13% at 300 kGy and 41.61% at 450 kGy). The reduction of tensile strength after EB irradiation is an indication of the degradation suffered by the NR/PS composites. 

Elongation at break is related with the resistance of material to shape changes until the fissures are formed. As can be seen in Figure 3, before irradiation, the addition of PS leads to an improvement of the elongation at break from 69.38% to 78.88% for 10 ppm addition, respectively 81.25% for the case of 50 ppm addition. After irradiation, the elongation at break decreases, regardless of the radiation dose and type of mixture, a result that is associated with the increasing of cross-linking degree and appearance of degradation. The composite became less ductile due to the restriction of the polymer chains’ free movement [33,34]. Samples without PS presented the highest variations of elongation at break as the irradiation dose increased, from 54.84% at 150 kGy up to 68.73% at 450 kGy. The addition of PS makes the material more stable in the radiation field. The statement is supported by results that are as follows: for 10 ppm addition (C10), from 11.25% (at 150 kGy) up to 61.96% (at 450 kGy); for 20 ppm addition (C20), from 10.23% (at 150 kGy) up to 31.52% (at 450 kGy); for 30 ppm addition (C30), from 7.53% (at 150 kGy) up to 30.11% (at 450 kGy); for 40 ppm addition (C40), from 13.06% (at 150 kGy) up to 31.25% (at 450 kGy); for 50 ppm addition (C50), from 5.53% (at 150 kGy) up to 51.38% (at 450 kGy). This is an interesting result taking into account the opposite tendencies of elongation and tensile strength. From the presented results, it can be observed that the mixtures that contain high loadings of PS still present an elastic behavior at the irradiation dose of 150 kGy. The composite is not brittle and the existence of a good compatibility between the PS and polymeric matrix can be assumed, even in the case of 50 ppm addition. A good dispersion of PS particles in the polymeric matrix compensates for the incompatibility between the hydrophobic nature of natural rubber and hydrophilic nature of starch. A large surface of PS particles is exposed to natural rubber molecules, leading to a huge interfacial volume around the filler (PS) and also to the hardness increasing, as can be seen from Figure 1. The mechanic resistance is increased by synergistic effect, the elongation at break is improved, and the fracture of the material takes place only after a long elongation [35]. Over the irradiation dose of 150 kGy, this trend is no longer maintained, the effect being proved by the elongation reduction. So, the increasing of PS loading and the applying of irradiation treatment leads to the decreasing of elongation, an effect associated with the appearance of degradation.

### 3.2. Sol-Gel and Cross-Link Density

**Figure 4:**
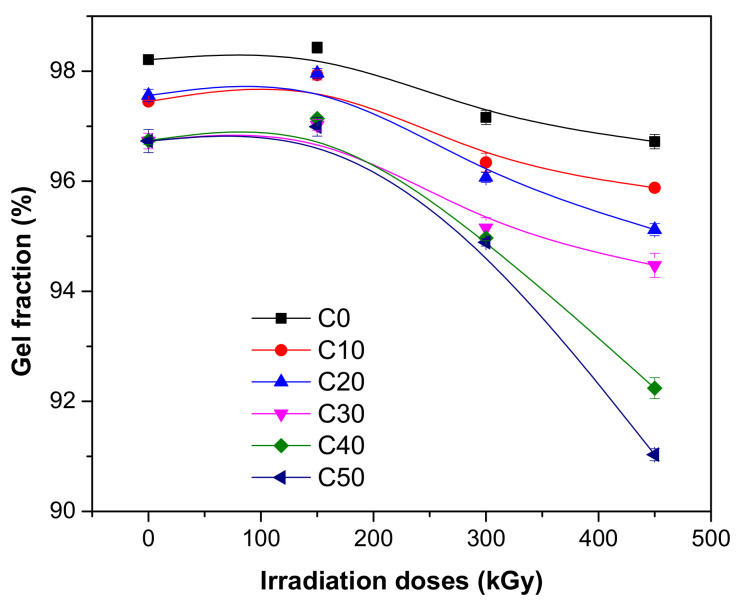
Gel fraction variation as a function of EB irradiation dose and PS amount.

**Figure 5:**
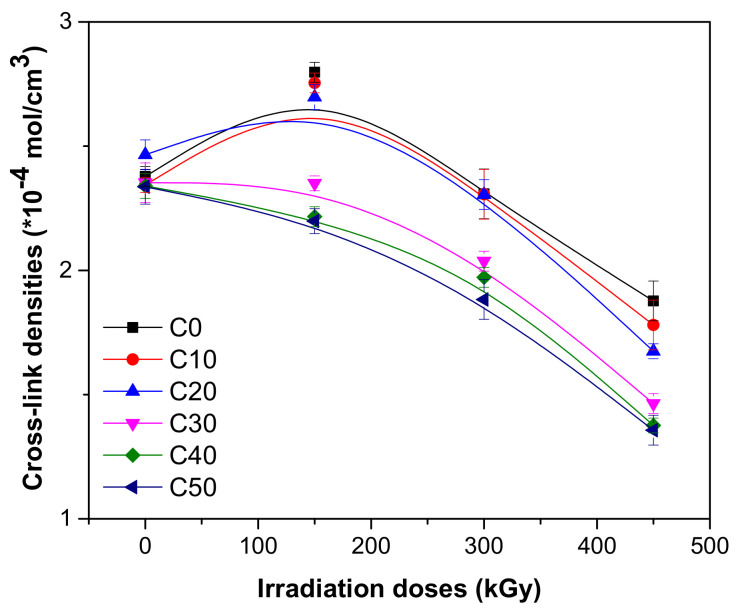
Cross-link density variation as a function of EB irradiation dose and PS amount.

The influence of PS loading and irradiation dose on gel fraction and cross-link degree was investigated and the results are presented in Figure 4 and Figure 5. 

The PS loading leads to a slow decreasing of both gel fraction and cross-link density before irradiation as follows: from 98.21% (C0) up to 96.73% for 50 ppm loading (C50) for gel fraction and from 2.377 × 10^−4^ mol/cm^3^ (C0) up to 2.3366 × 10^−4^ mol/cm^3^ for 50 ppm loading (C50) for cross-link density. The variations are quite small, 1.51% for gel fraction and 1.73% for cross-link density, a fact that can be explained by the changing of the phase structure of polymer matrix by introducing the filler. These results, correlated with those obtained after mechanical tests show that the composites before being subjected to EB irradiation present mechanical integrity and are not hard or brittle [36,37].

As seen in Figure 4 and Figure 5 at the irradiation dose of 150 kGy, samples without and with 10 and 20 ppm of PS, have showed increased gel fractions and cross-link densities. As the irradiation dose increased to 300 and 450 kGy, as was expected, the values of both properties decreased, especially for samples with high PS loading (C30, C40, and C50). At the irradiation dose of 300 kGy, the gel fraction reduction was 1.06% for C0 to 1.90% for C50 and at 450 kGy was 1.52% for C0 to 5.89% for C50. The cross-link density reduction was 2.93% for C0 to 19.42% for C50 at 300 de kGy and 21.06% for C0 to 41.93% for C50 at 450 kGy. For the results presented here, both cross-linking method with dibenzoyl peroxide and irradiation are responsible. Dibenzoyl peroxide decomposes at high temperatures (160 °C) and reacts with the NR matrix, and the mixture cross-linking is produced. Interaction between dibenzoyl peroxide and starch leads to the diminishing of hydroxyl groups and to the reduction of strong hydrophilic character of starch. The compatibility between dibenzoyl peroxide and starch is improved, a fact that is manifested in the properties of the composite [38]. In order to improve the interactions at the interface between the polymeric matrix and filler, was used starch in plasticized form (PS). Glycerine, used for starch plasticization, acts also as an anti-aggregation agent for starch particles [39,40,41]. In the structure of cross-linked NR/PS composite are found some types of chemical groups that can be modified (degraded) by EB irradiation as follows: C-O-C group that appears when PS is grafted on NR macromolecules [42], the glycosidic group of the starch molecule [42,43,44], and groups from NR as =CH_2_; -CH=CH_2_; -HC=CH-, or N–H [45,46]. The variation tendency of gel fraction and cross-link density, a small increase at 150 kGy, followed by a dramatic decreases with the irradiation dose increasing, shows that the cross-linking processes are accompanied by processes of cleavage of chemical bonds, i.e., degradation.

### 3.3. The Cross-Link and Chain Scission Yields

**Figure 6:**
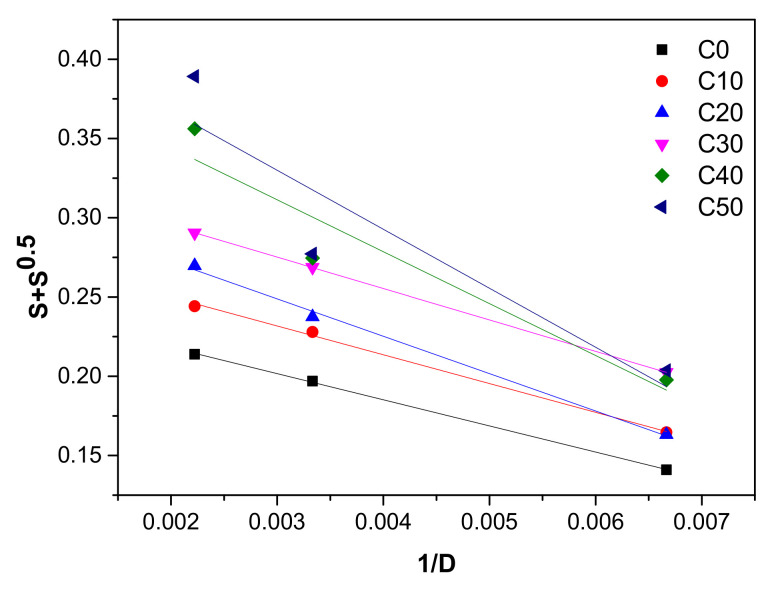
Charlesby–Pinner plots of NR/PS composites.

In order to determine the proportion between the cross-linking and scission, were done plots of *S* + *S*^1/2^ vs.1/absorbed dose (1/*D*) from the Charlesby–Pinner equation [25,47]. From results presented in Figure 6 was calculated the ratio p_0_/q_0_, whose values are presented in Table 3.

As seen in Table 3, the value of the *p_0_/q_0_* ratio increased as the PS amount in composite was higher. The increasing of *p_0_/q_0_* ratio is associated with the prevalence of scission reaction against cross-linking, being well known the fact that p_0_ represents the degradation degree (average number of chain scissions per monomer unit) and q_0_ the cross-link density.

In the case of polymers containing linear main chains, the predominant effects after treatments are chain branching and cross-linking. If the polymers have highly substituted quaternary atoms due to applied treatments, the molecular degradation or scissioning is the main effect. All the effects after irradiation with electron beam (cross-linking, grafting, chains scission) occur due to the presence of free radicals that are formed under the direct action of ionizing radiation upon the molecular structure, but also due to the newly formed species. Free radicals that are formed by C-H, C-O-C, C-C, or -C=C- bonds dissociation in the presence of H_2_ are responsible for the occurrence of cross-linking and chain branching reactions [6]. On the other hand, free radicals that are formed by dissociation of quaternary carbon bonds are more stable and do not migrate along the polymer chains due to the steric hindrance that favors their further evolution to chain scission and material degradation [6]. Thus, free radicals, cationic/anionic radicals, and excited molecules, very reactive species that are formed during irradiation, are responsible for the molecular structure changing of irradiated materials and the occurrence of cross-linking, chain branching, or scission. Cross-linking is associated with insoluble three-dimensional polymer networks characterized by high values of cross-linking degree and gel fraction, while scission is associated with degradation and characterized by decreased cross-linking degree and molecular weight. During irradiation, all these phenomena coexist and the occurrence of one or other depends on initial structure and morphology of polymer, yet also on the irradiation conditions (air, vacuum, inert gases). If the irradiation takes place in the air, the oxidative degradation occurs due to the presence of oxygen. This reaction competes with others in which oxidized functional groups such as carbonyl, peroxides, hydroperoxides, hydroxyl, and carboxyl do not generate the macromolecular oxidation that is associated with chain scission and molecular weight decreases [6].

In the case of NR/PS composites, the obtained results show that in the case of irradiation with 150 kGy, the grafting reaction occurred (the gel fraction and cross-linking degree values were high) and as the irradiation dose rose up to 450 kGy, chain scission (degradation) was dominant (the gel fraction and cross-linking degree values were lower).

### 3.4. Water Uptake

**Figure 7:**
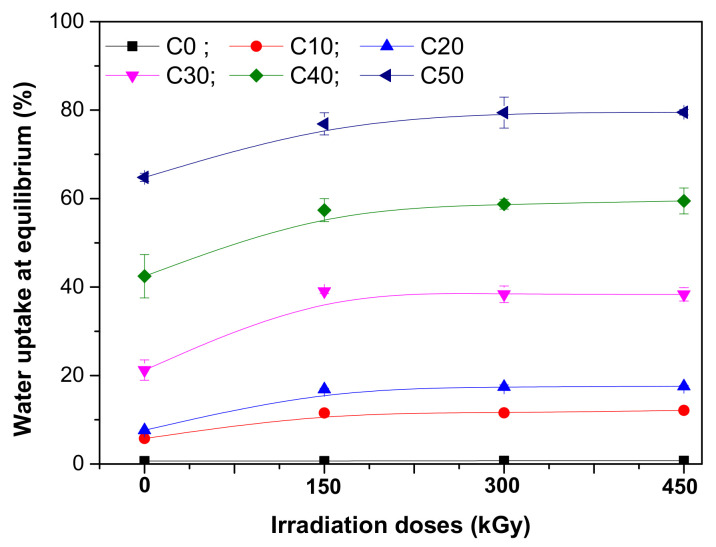
Water uptake at equilibrium of NR/PS samples.

Water uptake tests were done in order to demonstrate the occurrence and the influence of high hydrophilic polar groups during irradiation. The results are presented in Figure 7. 

The tested NR/PS composites were obtained both by peroxide vulcanization and electron beam irradiation. Generally, the structure of elastomers cured with peroxide is characterized by C-C cross-links (here due to the cross-linking of NR) and C-O links (here due to the grafting of PS on NR). Theoretically, it is difficult for the solvent molecule to penetrate C-C bonds due to their strong connection and high rigidity [48,49]. 

As seen in Figure 7, for the C0 sample (without PS), the water uptake percent was the smallest (0.69%) and the irradiation did not change it much (0.74% at 450 kGy). The non-irradiated mixtures in which the PS amount has varied between 10 and 50 phr, showed increased water absorption with the increasing of PS amount due to the hydrophilic nature of starch and of the large interfacial area between the starch and the natural rubber matrix, which facilitated the easier penetration of water molecules. Thus, the water uptake at equilibrium for C10 sample was of 11.52% while for C50 was 79.6%. For the composites based on elastomers and natural polymers, as for those based on NR and PS, the water absorption depends mainly on the water-soluble or hygroscopic components incorporated in the matrix that act as a semipermeable membrane, but also on the cross-linking degree because the adhesion between the fiber and the matrix is an important factor in determining the absorption behavior of a composite [35]. In the case of C10-C50 samples subjected to EB irradiation, the water uptake at equilibrium increased with the irradiation dose increasing, a possible explanation being the decreasing of C-C bonds number per unit of volume with the irradiation dose increasing, indicating a decreasing in cross-link density (Figure 5) and increasing of chain scissions (Figure 6 and Table 3). The destruction of C-C bonds in NR leads mainly to the macromolecular chain scission, while the destruction of C-O bonds in the NR/PS composite leads to the scission of grafting bonds [50]. The binding energies in C-C and C-O bonds are comparable (346 and 358 kJ, respectively), so we can conclude that both phenomena may equally appear during the irradiation process, leading to the composites’ degradation. Also, the materials irradiation in air generates free radicals and oxidized functional groups, and the oxidized macromolecules can suffer chains scissions and, as a consequence, the materials will present low molecular weights [6]. On the other hand, starch is a mixture of linear and branched components [51]. The EB irradiation of PS generates free radicals able to induce molecular changes and starch molecule fragmentation. Thus, the glycosidic bonds are decomposed in starch granules leading to macromolecule decomposition and the appearance of macromolecules with smaller chains [14,52,53]. All these correlated phenomena are responsible for starch degradation due to EB irradiation.

### 3.5. Weight Loss in Solvents (Toluene and Water)

**Figure 8:**
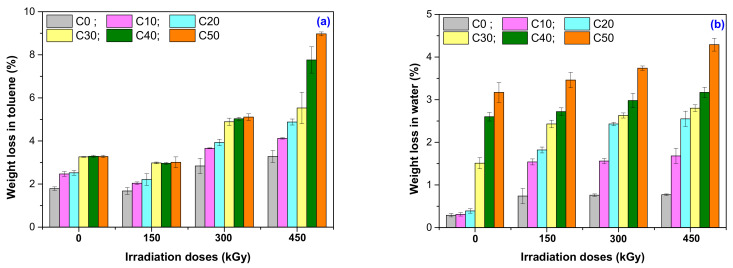
Weight loss of NR/PS samples in toluene (**a**) and water (**b**).

Another method to investigate the NR/PS composites degradation consists in the evaluation of weight loss by immersion, until the absorption at equilibrium is reached, in different solvents. The higher the mass of the sample at equilibrium, the higher the permeability of the solvent in the sample. Permeability is the one that determines the volume increasing of immersed samples, due to diffusion of the solvent molecules in the material structure. The samples that have reached the absorption equilibrium are dried until constant mass, then are weighed and if the mass is lower than initially, this means that some sample components were dissolved by the solvent [54]. The variation of weight loss in toluene and water for C0–C50 composites is presented in Figure 8a,b.

Correlating the results presented in Figure 4 and Figure 5 with those presented in Figure 8a, it can be seen that the weight loss in toluene is connected with the variation of gel fraction and cross-link density with the irradiation dose, these providing information about the cross-linking index (the average number of cross-links to which the initial polymer molecule is attached). Relation between the gel fraction and cross-linking density can be used also to determine the soluble fraction of the cross-linked polymer, useful to highlight the degradation process (the soluble fraction is a measure of degradation degree, i.e., the number of scissions) [55].

As seen in Figure 8a, even the non-irradiated sample C0 exhibits a weight loss of 1.79%, possibly due to the existence of some fractions of non-vulcanized NR. By applying the irradiation treatment, the weight loss increased up to 3.28%, due to the scissions in cross-linked elastomer, i.e., degradation. In the case of irradiated C0–C50 mixtures, the weight loss after the immersion in toluene increased both with the irradiation dose and PS amount. The non-irradiated NR/PS composites presented increased weight loss as the PS amount increased. Thus, the weight loss for C10 was 2.4% reaching up to 3.28% for C50. The weight loss for the irradiated sample C10 started from 2.4% at 150 kGy to 4.12% at 450 kGy, and for the irradiated sample C50 from 3.01% at 150 kGy to 8.97% at 450 kGy. The soluble fraction increased, a fact that also leads to the idea of installing the degradation process in the NR/PS composites. Because starch is a polysaccharide insoluble in water and organic solvents, the weight loss in toluene can be attributed to the degradation of NR by EB irradiation. 

The samples’ weight loss in water is presented in Figure 8b. The weight loss of C0 was the smallest, as was expected, due to the NR resistance to water action. It started from 0.28% for the non-irradiated sample and slowly increased with the irradiation dose to 0.76% at 150 kGy, to 0.75% at 300 kGy and to 0.75% at 450 kGy. The natural rubber contains in small percentages: proteins, fatty acids, resins, inorganic components composed by carbon, hydrogen, oxygen, and sulfur, but also polymeric chains formed by hundreds of individual amino acid residues connected to each other by peptide bonds. The latter are those that can be removed by immersion in water [56]. As can be seen from Figure 8a,b, the weight loss for non-irradiated C10-C50 samples was smaller in water than in toluene: 0.31% for C0 and 3.09% for C50, respectively. The same tendency can also be observed after irradiation. Even if the weight loss increased with the irradiation dose and PS amount, the values still remain under those that were found after the immersion in toluene: from 1.89% at 150 kGy to 2.06% at 450 kGy for C10 and from 3.33% at 150 kGy to 4.01% at 450 kGy for C50. Taking into account the NR resistance to water action, we can conclude that for the irradiated C10-C50 mixtures, the weight loss can be attributed to PS degradation. By EB irradiation, the scission of amylopectin (the PS branched component) and amylase (the PS linear component) bonds happened through the action of free radicals that are able to induce decomposition of the macromolecules [57,58]. 

### 3.6. Structural Investigations by Fourier Transform Infrared Spectroscopy (FTIR) Technique

**Figure 9:**
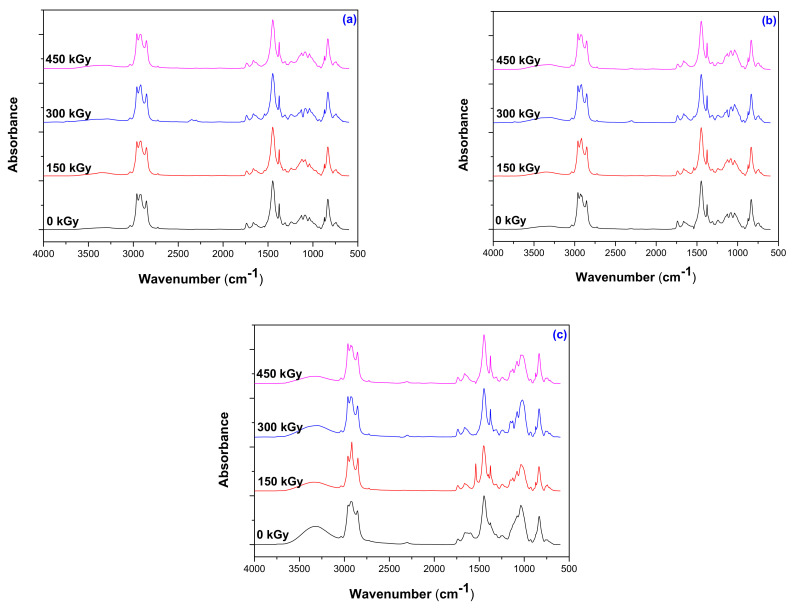
FTIR spectra for NR/PS composites (**a**) without PS (C0); (**b**) with 10 ppm of PS (C10); (**c**) with 50 ppm of PS (C50) in the range of 4000–600 cm^−1^.

The structure of the obtained composites was investigated through the Fourier Transform Infrared Spectroscopy (FTIR) technique in the range of 4000 to 600 cm^−1^. The results obtained for the irradiated samples without PS and with the lowest and the highest amount of PS are presented in Figure 9a–c.

As seen in Figure 9, some bands appeared to be modified by the reactions that took place during EB irradiation. The products that must be identified as being associated with the degradation are hydroperoxides, alcohols, aldehydes, epoxides, ketones, esters, and carboxylic acids [59,60]. There are some types of reactions that occur simultaneously in any material subjected to EB irradiation: cross-linking, chain branching, chain breaking with the formation of characteristic groups as methyl or tertbutyl due to the homolytic and heterolytic dissociations and oxidation with the formation of peroxides, alcohol groups, and carboxylic groups [61].

Spectra between 3800–3000 cm^−1^ realized for the irradiated and non-irradiates C0, C10, and C50 samples present a broad band between 3343–3317 cm^−1^ and 3306–3305 cm^−1^ due to the presence of proteins from NR [45]. After irradiation, C0 and C10 spectra present an increasing intensity of the wide curve between 3343–3317 cm^−1^ due to the formation of the hydroxyl group (-OH) as a result of degradation by oxidation [42,59]. The appearance of a wide peak is the result of inter- or intra-molecular hydrogen bonds between hydroxyl groups [59]. In C50 spectra, due to the high PS loading of 50 phr, the broad band between 3335 and 3350 cm^−1^ is most likely due the functional group –OH from starch and the band amplitude indicates the presence of the inter-molecular hydrogen bonds [62]. The peak absorption value of the –OH functional group decreases with increasing radiation dose due to the oxidation process [63]. For the same C0, C10, and C50 samples, the bands between 3036–3035 cm^−1^ correspond to the changes of carbon atoms’ substitution degree in the double bond (stretching vibration of =CH- in -CH=CH_2_ group from NR), a fact that is also connected with the degradation process [46].

For C0, C10, and C50 samples, the C=C group is of interest. Thus, we found between 1850–1650 cm^−1^ carbonyl compounds (R_2_C=O), above 1775 cm^−1^ active carbonyl groups as anhydrides or ring-carbonyl carbons, between 1750–1700 cm^−1^ simple carbonyl compounds as ketones, aldehydes, esters, or carboxyl, and below 1700 cm^−1^ amides or carboxylates functional group. 

In the region 1800–1500 cm^−1^, three bands can be observed: 1738–1733 cm^−1^, 1655–1651 cm^−1^, and 1549–1537 cm^−1^. FTIR spectra of C0, C10, and C50 present weak peaks in the region 1738–1733 cm^−1^, but with increasing of irradiation dose, especially at 300 kGy, C0 and C50 responses are modified due to the carbonyl groups (-C=O) and aldehyde (RCOH) that have been formed during degradation by EB irradiation [59,64]. It is no less true, that the specific bands of carbonyl groups (-C=O) can be attributed to peroxide decomposition used for the cross-linking of blends [65] and a decrease in their intensity with the increasing of PS loading and irradiation dose being connected with the cross-linking degree reduction by degradation.

Bands between 1655–1651 cm^−1^ are attributed to -C=C- stretching vibration in the NR structure, due to the hydroxyl stretching vibrations of absorbed water, carboxylate or conjugated ketone (>C=O) are also oxidative products derived from degradation [46]. Modified bands in the region 1655–1651 cm^−1^ are associated with the reduction of double bonds and oxidative products formation [66]. 

Two absorption bands characteristic of natural rubber can be observed in the region 1500–1300 cm^−1^: the first one due to the –CH_2_ deformation between 1448–1444 cm^−1^ and the second one due to the –CH_3_ asymmetric deformation between 1376–1374 cm^−1^ [67]. These bands’ modifications are more pregnant in the case of C10 and C50 and less for the C0 sample. The modified band between 1376–1374 cm^−1^ due to the plane bending vibration of O-H from starch highlights a structure modification through irradiation.

Absorptions that can be observed in the regions 1242–1239 cm^−1^ and 1154–1148 cm^−1^ can be attributed to the presence of some impurities in NR as proteins, lipids, amino acids and unstable peptides that can also be degraded by EB irradiation [67,68,69].

Bands located at 1088–1078 cm^−1^ (C-O-C deformation) and 965–928 cm^−1^ (OH flexion) are characteristic of polysaccharides and confirm the presence of amylose and amylopectin from starch. The band located between 1026 and 1024 cm^−1^ is associated with the amorphous component [62]. 

For C10 and C50 samples, the absorption band that has been found between 1087–1080 cm^−1^ (C-O-C) indicates that the PS grafting on NR structure [42] decreases with the irradiation dose increasing. Also, the band between 931–925 cm^–1^ can be attributed to the skeletal mode vibrations of α-(1-4) glycosidic linkage (C-O-C) and the one between 1039–1022 cm^–1^ to anhydrous glucose ring C-O stretch [43,44], both being modified by irradiation as follows: the first one slowly decreases and the second one increases for C0 and C10 and decreases for C50, both being associated with the composite degradation. Absorption registered in the region 872–833 cm^−1^ is characteristic for NR (cis-1,4 double bonds number in the polyisoprene chain) and slowly decreased with the irradiation dose increasing [46].

Thus, there are some modifications in the spectra obtained for the NR/PS composites that confirm the oxidative degradation by irradiation with doses between 150 and 450 kGy as follows: the decreasing of double bonds number in the NR chain (cis-1,4) observed between 872–833 cm^−1^, the increasing of glycosidic bond between 931–925 cm^−1^ near the decreasing of C-O-C absorption band in the region 1087–1080 cm^−1^ (specific to the PS grafting on NR), the presence of the –OH group between 3343–3317 cm^−1^, and the appearance of some weak bands between 1738–1733 cm^−1^ specific for –C=O and RCOH. 

The decreasing of *p_0_/q_0_* ratio and implicitly decreasing of cross-linking in favor of degradation are highlighted by the modification of the absorption band in the regions 1448–1444 cm^−1^, 1376–1374 cm^−1^, and 1655–1651 cm^−1^, and are attributed to –CH_2_ deformation, –CH_3_ asymmetric deformation, -C=C- stretching vibration in the NR structure, OH stretching vibrations of absorbed water, and carboxylate or conjugated ketone (>C=O). All of these are oxidative products resulted as a consequence of the degradation induced by EB irradiation.

### 3.7. Morphological Investigations by Scanning Electron Microscopy (SEM) Technique

**Figure 10:**
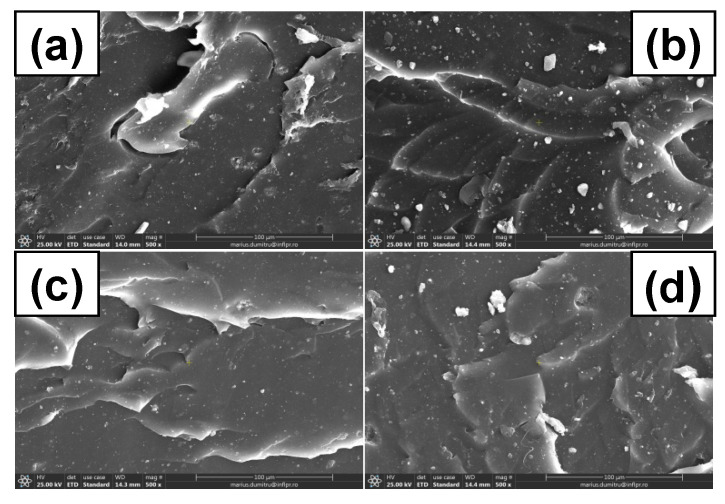
C0 SEM micrographs at magnification of 500: (**a**) un-irradiated, (**b**) irradiated at 150 kGy, (**c**) irradiated at 300 kGy, (**d**) irradiated at 450 kGy.

**Figure 11:**
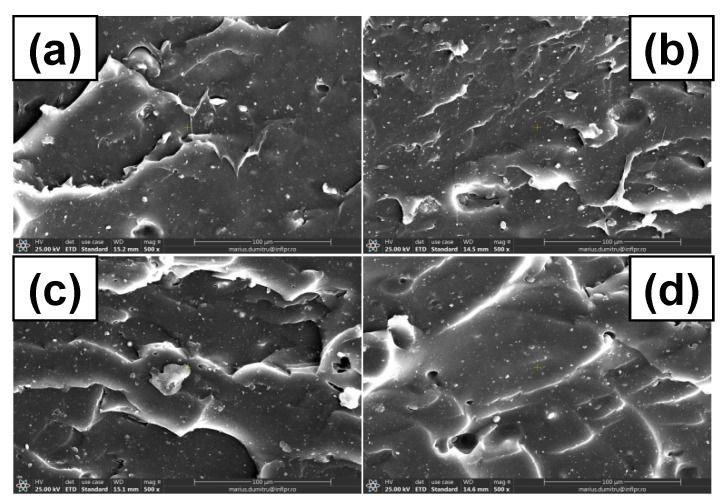
C10 SEM micrographs at magnification of 500: (**a**) un-irradiated, (**b**) irradiated at 150 kGy, (**c**) irradiated at 300 kGy, (**d**) irradiated at 450 kGy.

**Figure 12:**
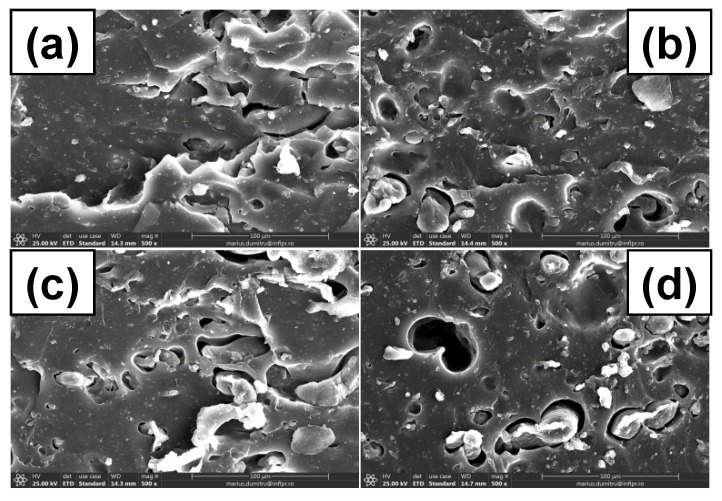
C50 SEM micrographs at magnification of 500: (**a**) un-irradiated, (**b**) irradiated at 150 kGy, (**c**) irradiated at 300 kGy, (**d**) irradiated at 450 kGy.

The surface morphological changes of C0, C10, and C50 samples before and after irradiation were evaluated by SEM analysis and some of the most representative images are presented in Figure 10, Figure 11 and Figure 12. Images were captured on fractured surfaces after tensile tests, on samples immersed in toluene in order to remove any split fragments or un-reacted materials.

In Figure 10, are presented the micrographs of C0 sample before and after irradiation at 150, 300, and 450 kGy. The surface homogeneity and smoothness that can be observed through the fracture are characteristic for a resistant material [70]. Small particles that can be observed may come from the vulcanization additives and imperfections of irradiated and non-irradiated specimens could be caused by the presence of some impurities remained after immersion in toluene. However, there are no significant differences between the SEM sample surfaces presented in Figure 10. Irrespective of the irradiation dose, no cracks or micro-cracks, holes, or rough areas appear on C0 surfaces.

In Figure 11a and Figure 12a, are presented SEM images of non-irradiated C10 and C50 samples. It can be seen that due to the cracking that have been formed after tensile stress, the composites present a porous surface, typical starch ball-like structure, which means that starch and NR matrix are compatible and the images do not show smooth structures as in Figure 10a [71]. There are some apparent holes on the composite fracture surface, especially in the case of C50, which suggest a weaker interaction between the starch and NR compared to C10 [72]. On the C10 SEM image, the uniformity and smoothness of the surface can be observed, like in the case of C0, which corresponds to a good adhesion between PS and NR.

As the irradiation dose increased (Figure 11 and Figure 12b,c), some changes could be observed on the C10 and C50 surfaces. The phenomenon is due to the aging of the material through the oxidative degradation. At the irradiation dose of 150 kGy, the slow increasing of the cross-linking degree makes us think that the cross-linking reaction is still going on. After that, at 300 and 450 kGy, the cross-linking reaction becomes degradation with the formation of short fragments—oligomers that were than eliminated by the immersion in water and toluene. 

The holes observed in Figure 11 and Figure 12 at the irradiation dose of 300 and 450 kGy are due, on the one hand, to the poor adhesion between the filler and the elastomeric matrix, especially for the C50 mixture, but also to the formation of small molecule of compounds, which appear after degradation by irradiation. All of these are very well correlated with the results obtained in mechanical tests (hardness increasing, tensile strength, and elongation decreasing with the irradiation dose increasing over 150 kGy). With the irradiation dose increasing, the surfaces of both C10 and C50 samples present cracks and micro pores [70,73]. This result suggests the decreasing of the adhesion at the interface as the irradiation dose increases, which leads to material degradation. 

## 4. Conclusions

Degradation of polymeric composites based on natural rubber (NR) and plasticized starch (PS) by electron beam (EB) irradiation in the dose range of 150 and 450 kGy was investigated. The NR/PS composites containing PS in amounts between 10 to 50 phr (C10-C50) were previously obtained by peroxide cross-linking. The Charlesby–Pinner equation was used to appreciate the cross-linking and the chain scission reaction yields. The increasing of *p_0_/q_0_* ratio with the PS amount and irradiation dose was associated with the prevalence of scission reaction specific for degradation process as against cross-linking. At the irradiation dose of 150 kGy, especially for NR and C10 samples, the slowly increasing cross-linking degree and tensile strength made us think that the cross-linking reaction continues, probably being unfinished in peroxide cure. At 300 and 450 kGy, the cross-linking reaction degraded with the formation of short fragments—oligomers that were eliminated by the immersion in water and toluene. The small amount of PS in C10 made its mechanical and sol-gel behavior to be closer to that of NR compared to that of other samples (C20-C50) irradiated at 300 and 450 kGy. As the irradiation dose increased up to 450 kGy, some changes on the C10 and C50 surfaces due to the aging of the material through the oxidative degradation by irradiation were emphasized by SEM investigation. 

Modifications in the FTIR spectra of NR/PS composites confirm the oxidative degradation at the irradiation dose of 300 and 450 kGy as follows: the decreasing of double bonds number in the NR chain (cis-1,4) observed between 872–833 cm^−1^, the increasing of glycosidic bond between 931–925 cm^−1^ near the decreasing of C-O-C absorption band in the region 1087–1080 cm^−1^, the presence of the –OH group between 3343–3317 cm^−1^, and the appearance of some weak bands between 1738–1733 cm^−1^ specific for –C=O and RCOH. 

The evolution of the p_0_/q_0_ ratio associated with the –CH_2_ deformation, –CH_3_ asymmetric deformation, -C=C- stretching vibration in the NR structure, OH stretching vibrations of absorbed water, and carboxylate or conjugated ketone (>C=O) in the FTIR spectra confirmed the appearance of oxidative products resulted as a consequence of the degradation induced by EB irradiation.

According to the current trends that request to replace the conventional hazardous active fillers with environmentally friendly fillers, PS can be a promising alternative. Also, EB irradiation can be a viable solution for rubber product degradation.

## Figures and Tables

**Table 1:** Physical and chemical characteristics of raw materials.

Material	Properties
Natural rubber Crepe 1X, from Sangtvon Rubber Ltd., Nakhon Si Thammarat, Thailand	Mooney viscosity: 67.64 ML1+4 at 100 °C, volatile materials content: 0.5%, nitrogen content: 0.4%, ash: 0.25%, impurities content: 0.026%
Soluble potato Starch, from Lach-Ner, Neratovice, Czech Republic	Water insoluble substances: 0.28%, loss on drying: 16.9%, easily biodegradable: BOD5—0.6 g/g and COD—1.2 mg/g
Glycerine, from SC Chimreactiv SRL, Bucharest, Romania	Density: 1.26 g/cm^3^, purity: 99.5%, free acidity: 0.02% Used for plasticized starch obtaining
IPPD antioxidant (4010 NA) N-isopropyl-N-phenyl-phenylene diamine, from Dalian Richon Chem Co. Ltd., Dalian, China	Molecular mass: 493.6374 g/mol, purity: 98%, Used as antioxidant
Perkadox 40 dibenzoyl peroxide, from AkzoNobel Chemicals, Deventer, Netherlands	Density: 1.60 g/cm^3^, active oxygen content: 3.8%, peroxide content: 40%, pH 7 Used as cross-linking agent
TMPT DL 75 Luvomaxx-trimethylolpropane trimethacrylate, from Lehmann & Voss & Co Hamburg, Germany	Density 1.36 g/cm^3^, ash: 22%, pH 9.2, active ingredient: 75 ± 3%. Used as co-agent curing

**Table 2:** The recipes used in obtaining composites.

Ingredients (phr)	Mixtures Codes					
	NR	NR-10	NR-20	NR-30	NR-40	NR-50
Natural rubber (NR)	100	100	100	100	100	100
Starch	0	10	20	30	40	50
Glycerine	0	6	12	18	24	30
Dibenzoyl Peroxyde	8	8	8	8	8	8
TMPT	3	3	3	3	3	3
IPPD Antioxidant	1	1	1	1	1	1

**Table 3:** Compositional characteristics and p_0_/q_0_ ratio for NR/PS composites.

PS Amount in NR/PS Composites	C0	C10	C20	C30	C40	C50
*p_0_/q_0_* ratio	0.2514	0.2862	0.3195	0.3347	0.4096	0.4412

## Data Availability

The data presented in this study are available on request from the corresponding author.
